# Knowledge, attitudes, and practices among medical students toward depression management: a cross-sectional study in China

**DOI:** 10.3389/fpubh.2024.1429943

**Published:** 2024-10-07

**Authors:** Wei Zhang, Xiaolin Wu, Mi Li, Guoli Wang, Yan Liu, Xin Zhang, Linxuan Zhang

**Affiliations:** ^1^School of Medical Imaging, Binzhou Medical University, Yantai, China; ^2^Orthopedics Department, The Affiliated Hospital of Qingdao University, Qingdao, China; ^3^Shandong College of Traditional Chinese Medicine, Yantai, China; ^4^School of Traditional Chinese Medicine, Binzhou Medical University, Yantai, China

**Keywords:** knowledge, attitudes, practices, depression, medical students, cross-sectional study

## Abstract

**Background:**

The prevalence of depression among university and college students in China is nearly one-quarter. This study aimed to investigate the knowledge, attitudes and practices (KAP) among medical students toward depression management. Depression, a significant public health issue, has a substantial impact on students, with a reported prevalence of 23.8% among university and college students in China.

**Methods:**

This web-based cross-sectional study was conducted between January 2023 and October 2023 at three medical universities in China. A self-administered questionnaire collected demographic information and assessed medical students’ KAP on depression management.

**Results:**

A total of 632 participants were enrolled in this study from three medical universities. Among them, 383 (60.60%) were female, and the participants were aged 20.17 years on average (SD ± 1.80). Most of their family members (521, 82.44%) were not in the medical profession. Using a validated 15-point knowledge scale, a 50-point attitude scale, and a 30-point practice scale, which covered areas such as symptom recognition, treatment approaches, and stigma related to depression, the mean knowledge, attitudes and practices scores were 10.55 ± 3.36 (possible range: 0–15), 41.72 ± 4.45 (possible range: 10–50) and 19.79 ± 5.44 (possible range: 6–30), respectively. Males had lower knowledge (*p* = 0.003). Only children had higher practice (*p* = 0.034). Urban residents had higher attitudes (*p* = 0.046). Higher income linked to better practice (*p* = 0.047). Freshmen scored higher across all KAP (*p* < 0.05). Medical family background linked to better knowledge (*p* = 0.005). The attitude scores were correlated with the practice scores (*r* = 0.403, *p* = 0.004). The structural equation model demonstrated that knowledge had direct effects on attitude and practice, as indicated by a path coefficient of 0.725 (*p* < 0.001) and 0.370 (*p* = 0.001), respectively. Furthermore, attitude had direct effects on practices, with a path coefficient of 0.509 (*p* < 0.001).

**Conclusion:**

The study revealed that medical students possessed sufficient knowledge and demonstrated active attitudes but exhibited limited practical skills in managing depression. In investigating the KAP of medical students, it is essential to integrate specific practical applications, such as role-playing scenarios and case studies, into the curriculum. These methods will emphasize the importance of knowledge, foster positive attitudes, and promote peer collaboration to enhance practical skills in depression management.

## Introduction

Depression is a significant global public health concern, particularly in China, where it has substantial epidemiological implications (1). Globally, an estimated 322 million individuals grapple with depressive disorders, with a 3.6% prevalence of 12-month depression in the general Chinese population and a worrisome 23.8% prevalence among university and college students (2, 3). This high prevalence underscores depression’s status as a psychiatric disorder characterized by elevated rates of morbidity and mortality (4). The global prevalence of underdiagnosed and undertreated depression, affecting a substantial 40 to 85% of individuals worldwide, raises significant concerns (5). Moreover, stigma surrounding depression remains pervasive, extending even to medical students (3). Furthermore, depression and its complications, such as suicide and social and academic impairment, among a large proportion of young people in the country, could have a negative impact on the nation at large (6). Medical students represent the prospective medical doctors who will serve as the cornerstone of the nation’s healthcare workforce (7). The role of medical education in molding the perspectives and approaches of these future healthcare professionals toward various medical conditions, including depression, is pivotal (8, 9). Given their forthcoming roles as frontline healthcare providers, the comprehension of depression among medical students takes on heightened importance. Their knowledge and attitudes concerning the diagnosis, treatment, and management of depression hold substantial sway over their interactions and practices when caring for individuals grappling with this condition. Currently, there exists a notable research gap in the exploration of medical students’ knowledge, attitudes, and practices toward the prevention and treatment of depression.

Knowledge, Attitudes, and Practices (KAP) studies serve as a widely utilized research methodology to assess the comprehension and behaviors of specific populations regarding a particular subject. Within the medical field, KAP studies have proven effective in evaluating healthcare professionals’ awareness, attitudes, and practices pertaining to various medical conditions and treatment approaches (10–12). Therefore, this study aimed to fill the research gap by investigating the KAP of medical students toward the management of depression. By understanding medical students’ perspectives and behaviors toward depression, this study seeks to identify potential areas for improvement in depression-related medical education and enhance the quality of care provided to individuals suffering from this mental health disorder.

## Methods

### Study design and participants

This cross-sectional survey was conducted between January 2023 and October 2023 at Binzhou Medical University, the Affiliated Hospital of Qingdao University and Weifang Medical University. The study was ethically approved by the Ethics Committee of Binzhou Medical University (Approval no. 2023–316), the Ethics Committee of the Affiliated Hospital of Qingdao University (Approval no. QYFY WZLL 28213) and Weifang Medical University and informed consent was obtained from the study participants.

Medical students from three sites were included in this study. Those possessing a documented personal history of depression or other mental health disorders (as their cognitive outlook, attitudes, and practices may be influenced differently) or those facing hindrances in completing the study questionnaire or participating in interviews attributable to language barriers, cognitive impairments, or other communication-related challenges were excluded.

### Questionnaire

The questionnaire was developed with guidance from relevant literature on depression management (13–15). The first draft was pilot tested on a small scale (*n* = 16), resulting in a Cronbach’s alpha coefficient value of 0.836, indicating good internal consistency.

The final questionnaire was in Chinese and had four parts: demographic information (13 items), knowledge (15 items), attitudes (10 items), and practices (8 items). For knowledge, participants received 1 point for each correct answer and 0 points for others, resulting in a possible score range of 0 to 15. Attitude items used a five-point scale, with responses ranging from 5 points for “strongly agree” to 1 point for “strongly disagree,” resulting in a possible score range of 10 to 50. The practice items also used a five-point scale, where “always/very consistent” received 5 points and “never/very inconsistent” received 1 point, since P7 and P8 were not scored but only included for descriptive purposes, the possible score range of practices is 6 to 30.

The data were collected using an online questionnaire hosted on Sojump^1^. The online questionnaire was distributed through various social media platforms, including WeChat, internet forums, and web links. To prevent duplication, an IP restriction was implemented, ensuring that each survey could only be completed once per unique IP address.

### Statistical analysis

Statistical analysis was conducted using STATA 17.0 (Stata Corporation, College Station, TX, United States) and AMOS 24.0 (IBM, NY, United States). Continuous variables were described using mean ± standard deviation (SD), and between-group comparisons were performed using *t*-tests or analysis of variance (ANOVA). Categorical variables were presented as *n* (%). Pearson’s analysis was employed to assess the correlations between knowledge, attitude, and practice scores. The structural equation model (SEM) was employed to examine the relationships among knowledge, attitudes, and practices. The hypotheses were as follows: (1) knowledge had direct effects on attitudes, (2) knowledge had direct effects on practices, and (3) attitudes had direct effects on practices. The model fitting was evaluated with CMIN/DF (Chi-square fit statistics/degree of freedom), RMSEA (root mean square error of approximation), IFI (incremental fix index), TLI (Tucker–Lewis index) and CFI (comparative fix index). Two-sided *p* < 0.05 were considered statistically significant in this study.

## Results

A total of 632 valid questionnaires were enrolled in the final analysis, with a validity rate of 81.55%. Among them, 383 (60.60%) of the participants were females, with a mean age of 20.17 ± 1.80, 457 (72.31%) were the only child, 411 (65.03%) lived in rural areas, 596 (94.30%) were currently studying in an undergraduate degree programs, 371 (58.70%) of the fathers and 431 (68.20%) of the mothers of the participants had a junior high school education or less. In addition, most of the family members of the participants (521, 82.44%) were not in the medical profession (Table 1).

**Table 1:** Demographic characteristics and KAP scores.

	*N* (%)	Knowledge		Attitudes		Practices	
		Mean ± SD	*P*	Mean ± SD	*P*	Mean ± SD	*P*
Total	632	10.55 ± 3.36		41.72 ± 4.45		19.79 ± 5.44	
Gender			0.003		0.791		0.096
Male	249 (39.40)	10.06 ± 3.69		41.78 ± 4.37		20.24 ± 6.09	
Female	383 (60.60)	10.87 ± 3.09		41.68 ± 4.51		19.50 ± 4.97	
Age (years)	20.17 ± 1.80						
Only child			0.124		0.294		0.034
Yes	175 (27.69)	10.89 ± 3.25		42.02 ± 4.28		20.53 ± 5.53	
No	457 (72.31)	10.43 ± 3.40		41.60 ± 4.51		19.51 ± 5.39	
Residence			0.200		0.046		0.696
Rural	411 (65.03)	10.43 ± 3.46		41.46 ± 4.52		19.73 ± 5.44	
Urban	221 (34.97)	10.79 ± 3.16		42.20 ± 4.30		19.90 ± 5.47	
*Per capita* income (CNY)			0.554		0.077		0.047
<2,000	100 (15.82)	10.35 ± 3.50		41.37 ± 4.85		18.36 ± 6.15	
2,000–5,000	264 (41.77)	10.70 ± 3.26		41.80 ± 4.27		20.01 ± 5.22	
5,000–10,000	180 (28.48)	10.56 ± 3.43		41.61 ± 4.70		20.05 ± 5.34	
10,000-20,000	68 (10.76)	10.07 ± 3.54		41.40 ± 4.08		19.87 ± 4.97	
>20,000	20 (3.16)	11.20 ± 2.71		44.40 ± 2.76		21.45 ± 6.20	
University type			0.441		0.518		0.084
General university	627 (99.21)	10.56 ± 3.35		41.71 ± 4.45		19.82 ± 5.39	
Double first-class university	5 (0.79)	9.40 ± 4.72		43.00 ± 4.80		15.60 ± 10.04	
Current academic level			0.552		0.022		0.006
College diploma	4 (0.63)	12.25 ± 0.50		46.00 ± 0.82		28.00 ± 3.37	
Bachelor’s degree	596 (94.30)	10.51 ± 3.40		41.61 ± 4.47		19.83 ± 5.32	
Master’s degree	28 (4.43)	11.00 ± 2.76		42.86 ± 4.01		18.11 ± 5.74	
Doctoral degree	4 (0.63)	11.75 ± 0.50		46.00 ± 0.00		17.50 ± 13.30	
Father’s education level			0.611		0.620		0.630
Junior high school or below	371 (58.70)	10.49 ± 3.39		41.60 ± 4.30		19.71 ± 5.49	
High school or vocational school	164 (25.95)	10.48 ± 3.40		41.84 ± 4.76		19.88 ± 5.27	
College or bachelor’s degree	88 (13.92)	11.00 ± 3.13		41.81 ± 4.62		20.15 ± 5.09	
Master’s degree or above	9 (1.42)	10.33 ± 3.77		43.44 ± 3.40		17.78 ± 9.40	
Mother’s education level			0.556		0.470		0.182
Junior high school or below	431 (68.20)	10.47 ± 3.39		41.53 ± 4.51		19.71 ± 5.38	
High school or vocational school	137 (21.68)	10.71 ± 3.19		42.12 ± 4.37		19.88 ± 5.70	
College or bachelor’s degree	55 (8.70)	10.98 ± 3.48		42.18 ± 4.32		20.71 ± 4.38	
Master’s degree or above	9 (1.42)	9.67 ± 3.77		41.78 ± 3.60		16.56 ± 8.89	
Grade			0.029		0.044		0.001
Freshman	237 (37.50)	10.92 ± 2.85		41.78 ± 4.25		20.68 ± 5.06	
Sophomore	192 (30.38)	10.42 ± 3.60		41.80 ± 4.56		19.63 ± 5.58	
Junior	129 (20.41)	10.10 ± 3.84		41.33 ± 4.81		18.63 ± 6.06	
Senior	22 (3.48)	11.91 ± 1.60		44.27 ± 2.33		17.09 ± 4.51	
Fifth year or above	52 (8.23)	9.94 ± 3.62		40.98 ± 4.44		20.35 ± 4.50	
Specialty			0.105		0.068		0.621
Pharmaceutical sciences	65 (10.28)	11.00 ± 2.87		41.85 ± 4.43		20.38 ± 5.56	
Nursing sciences	1 (0.16)	12.00		46.00		18.00	
Medical laboratory sciences	2 (0.32)	12.00 ± 0.00		46.00 ± 0.00		20.00 ± 1.41	
Clinical medicine (general)	57 (9.02)	11.53 ± 2.22		43.19 ± 3.71		18.70 ± 6.02	
Public health management	2 (0.32)	13.00 ± 1.41		42.00 ± 5.66		22.00 ± 4.24	
Medical technology or related specialties	505 (79.91)	10.37 ± 3.51		41.51 ± 4.51		19.83 ± 5.37	
Recent semester GPA ranking			0.081		0.429		0.948
Top 25%	130 (20.57)	10.65 ± 3.42		41.76 ± 5.10		19.72 ± 6.08	
26–50%	189 (29.91)	10.55 ± 3.25		41.90 ± 4.07		19.99 ± 5.44	
51–75%	132 (20.89)	10.45 ± 3.53		41.27 ± 4.46		19.57 ± 4.90	
Top 75%	55 (8.70)	9.49 ± 4.22		41.09 ± 4.50		19.49 ± 5.93	
Not assessed yet	126 (19.94)	11.03 ± 2.74		42.13 ± 4.26		19.92 ± 5.12	
Family members currently in the medical profession			0.005		0.053		0.143
Yes	111 (17.56)	11.36 ± 2.50		42.46 ± 4.10		20.48 ± 5.36	
No	521 (82.44)	10.38 ± 3.49		41.56 ± 4.51		19.64 ± 5.45	

The knowledge scores of participants ranged from a minimum of 0 to a maximum of 15, with a mean score of 10.55 ± 3.36. Attitude scores varied between 10 and 50, with an average of 41.72 ± 4.45. Practice scores spanned from 6 to 30, averaging 19.79 ± 5.44. The results indicate several statistically significant associations between demographic characteristics and KAP scores. Males exhibited lower knowledge levels compared to females (*p* = 0.003), while individuals who are not only children showed lower practice scores than those who are (*p* = 0.034). Urban residents had higher attitude scores compared to rural residents (*p* = 0.046). Participants with a *per capita* income greater than 20,000 CNY demonstrated better practice scores compared to those with lower incomes (*p* = 0.047). Furthermore, students with a college diploma had higher attitude (*p* = 0.022) and practice scores (*p* = 0.006) than those with other academic levels. Freshman students had significantly higher knowledge (*p* = 0.029), attitude (*p* = 0.044), and practice scores (*p* = 0.001) compared to other grades. Additionally, participants with family members in the medical profession had higher knowledge scores (*p* = 0.005) than those without family members in the medical profession (Table 1).

The distribution of knowledge dimensions revealed that the scores for the top three knowledge items were as follows: “The treatment of depression should adhere to a personalized medication approach, tailored to the individual patient’s circumstances.” (K13) with a score ranging from 0 to 1, where the highest score was 0.88, “A comprehensive assessment of cognitive functioning in individuals with depression requires both subjective and objective cognitive measurements or the use of comprehensive test tools.” (K11) with a maximum score of 0.87, and “Comorbid anxiety disorders often accompany depression in patients, necessitating careful differentiation in clinical practice.” (K6) with a top score of 0.86. The three knowledge items with the lowest scores were “Cognitive symptoms in depression typically arise only during acute episodes.” (K4), scoring as low as 0.29, “Combination therapy with antidepressant medication is the preferred approach for the treatment of depression, regardless of disease severity, with monotherapy not being considered.” (K10), with a score of 0.28, and “Major depressive disorder is the most common psychiatric disorder, with depression being its shorthand.” (K1), scoring only 0.07 (Table 2).

**Table 2:** Knowledge.

	Correctness rate *N* (%)
K1: Major depressive disorder is the most common psychiatric disorder, with depression being its shorthand.	46 (7.28)
K2: Clinical manifestations of depression are diverse, encompassing emotional, physical, and cognitive dimensions.	523 (82.75)
K3: Difficulties in concentration, memory impairment, and decreased information processing speed may all manifest in individuals with depression as cognitive symptoms.	536 (84.81)
K4: Cognitive symptoms in depression typically arise only during acute episodes.	185 (29.27)
K5: Physical symptoms during depressive episodes can involve various organ systems, commonly presenting as early awakening, reduced appetite, and weight loss.	551 (81.49)
K6: Comorbid anxiety disorders often accompany depression in patients, necessitating careful differentiation in clinical practice.	547 (86.55)
K7: In the treatment of depression, the ultimate goal is the restoration of a patient’s social functioning and quality of life.	467 (73.89)
K8: In clinical practice, it is essential to discern whether depression in a patient is a secondary condition caused by substances or medications.	515 (81.49)
K9: The diagnosis of depression in patients should encompass a comprehensive assessment, including a medical history evaluation, psychiatric examination, assessment using relevant scales, neuroimaging for structural, functional, and metabolic changes, and so forth.	534 (84.49)
K10: Combination therapy with antidepressant medication is the preferred approach for the treatment of depression, regardless of disease severity, with monotherapy not being considered.	178 (28.16)
K11: A comprehensive assessment of cognitive functioning in individuals with depression requires both subjective and objective cognitive measurements or the use of comprehensive test tools.	552 (87.34)
K12: Common guidelines for depression can be referenced, such as those provided by NICE, APA, WFSPB, among others.	476 (75.32)
K13: The treatment of depression should adhere to a personalized medication approach, tailored to the individual patient’s circumstances.	557 (88.13)
K14: Commonly used medications for the treatment of depression include tricyclic antidepressants and selective serotonin reuptake inhibitors, among others.	471 (74.53)
K15: Antidepressant medications should ideally be used as monotherapy, with combination therapy considered only in cases of treatment-resistant depression to enhance efficacy.	532 (84.18)

In this study, participants’ attitudes toward depression showed the following score ranges: 55.70% of the participants strongly agreed that the onset of depression is often insidious, and timely diagnosis and early intervention are important for the prognosis of the disease (A1), corresponding to a score of 0.56. Scores for the item “Depression is prone to recurrence and affects the quality of survival of depressed patients.” (A2) ranged from 0 to 1, with the highest being 0.59. Notably, “The progression of depression may trigger suicide attempts or death in patients, resulting in serious adverse consequences.” (A3) showed the highest score of 0.92. On the other hand, only 27.85% were neutral about the interest in learning about depression-related diagnosis and treatment through books, courses, literature, or the internet (A4), corresponding to a score of 0.28 (Table 3).

**Table 3:** Attitudes.

	Strongly agree *N* (%)	Agree *N* (%)	Neutral *N* (%)	Disagree *N* (%)	Strongly disagree *N* (%)
A1. Depression often has a concealed onset; timely diagnosis and early intervention are crucial for the prognosis of the condition.	352 (55.70)	238 (37.66)	42 (6.65)	0	0
A2. Depression tends to recur, and its high recurrence rate impacts the quality of life for individuals with depression. Therefore, it requires special attention during diagnosis and treatment.	371 (58.70)	222 (35.13)	38 (6.01)	1 (0.16)	0
A3. The progression of depression may lead to suicide attempts or deaths, resulting in severe consequences. Therefore, it should be taken seriously.	582 (92.09)	48 (7.59)	2 (0.32)	0	0
A4. In your academic and personal life, you have a strong interest in acquiring knowledge about the diagnosis and treatment of depression through books, courses, literature, and online resources.	239 (37.82)	209 (33.07)	176 (27.85)	7 (1.11)	1 (0.16)
A5. You acknowledge that different treatment approaches can have a significant impact on the prognosis of depression patients; hence, the competence of healthcare professionals plays a crucial role.	296 (46.84)	259 (40.98)	75 (11.87)	2 (0.32)	0
A6. You believe that there is a gap between medical students and clinical practitioners, leading to limited practical understanding of depression-related diagnosis and treatment methods.	231 (36.55)	251 (39.72)	118 (18.67)	31 (4.91)	1 (0.16)
A7. The diagnosis of depression is complex and multidimensional. Therefore, medical students should approach it from a professional perspective and tailor their in-depth learning based on their specific field of study. For instance, nursing students should focus on disease management, pharmacy students on appropriate medication and adverse reactions, and clinical psychology students on relevant assessment tools and diagnostic criteria.	285 (45.09)	266 (42.09)	78 (12.34)	3 (0.47)	0
A8. When confronted with depressive feelings stemming from stress in work or academic settings, it is essential to address them actively and seek timely counseling.	323 (51.11)	259 (40.98)	49 (7.75)	1 (0.16)	0
A9. You believe that if diagnosed with depression, it is the right course of action not to conceal it and to seek help from your surroundings promptly, while actively engaging in treatment.	299 (47.31)	268 (42.41)	61 (9.65)	3 (0.47)	1 (0.16)
A10. You hold the view that society should provide greater tolerance and support to individuals suffering from depression.	325 (51.42)	252 (39.84 = 7)	51 (8.07)	1 (0.16)	3 (0.47)

With regard to practices, only 20.41% of participants scored highly for consistently learning about depression through books and literature (P1), with scores ranging from 0 to 1, while 42.25% took the initiative to learn about depression assessment tools (P3), scoring up to 0.42. In terms of providing psychological support to people around them, the scores for the item “When people around them were found to have some tendency to depression, 61.55% would use the professional knowledge they learned to carry out psychological counseling for those people.” (P6), ranged up to 0.62 (Table 4).

**Table 4:** Practices.

	Always *N* (%)	Often *N* (%)	Sometimes *N* (%)	Occasionally *N* (%)	Never *N* (%)
P1. How often do you study depression-related knowledge through books and literature (e.g., common symptoms, diagnostic and treatment methods)?	66 (10.44)	63 (9.97)	189 (29.91)	211 (33.39)	103 (16.30)

	Very consistent *N* (%)	Somewhat consistent *N* (%)		Neutral *N* (%)	Somewhat Inconsistent *N* (%)		Very inconsistent *N* (%)	
P2. Are you consciously participating in depression-related elective courses or training (e.g., lectures or clinical internships)?	88 (13.92)	156 (24.68)		255 (40.35)	90 (14.24)		43 (6.80)	
P3. Are you consciously studying depression assessment scales, such as the SDS scale, PHQ-9 scale, and staying updated with the latest guidelines or expert consensus?	102 (16.14)	165 (226.11)		225 (35.60)	94 (14.87)		46 (7.28)	
P4. Do you pay more attention to the depressive tendencies or states of people around you?	113 (17.88)	236 (37.34)		197 (31.17)	63 (9.97)		23 (3.64)	
P5. Do you consciously use various mental assessment scales in daily life to assess your own or others’ depressive tendencies?	109 (17.25)	194 (30.70)		205 (32.44)	89 (14.08)		35 (5.54)	
P6. When you notice that classmates or family members around you have certain depressive tendencies, do you use your acquired professional knowledge to provide psychological counseling for them?	117 (18.51)	272 (43.04)		173 (27.37)	42 (6.65)		28 (4.43)	
	Cultivate a positive outlook on life	Maintain a regular exercise regimen	Widespread mental health education	Counselors/Schools paying increased attention to students’ mental well-being	Positive content promotion on online media	Seek support and confide in family and friends	Engage in reading or meditation practices	
P7.Effective Preventive Measures for Depression which you would consider: (Multiple Selections)	572(90.51)	552(87.34)	549(86.87)	431(68.20)	416(65.82)	470(74.37)	356(56.33)	
	Want to stay away from him/her	Cannot understand, feel confused	Want to offer help to the best of my ability	Feel indifferent, detached	Feel pity but powerless	Think he/she is joking	Think he/she is just seeking attention from others	Other
P8. If you hear that your classmates around you are suffering from depression, how would you react? (Multiple choices)	42(6.65)	60(9.49)	551(87.18)	46(7.28)	188(29.75)	31(4.91)	33(5.22)	25(3.96)

Pearson’s analysis showed that significant positive correlations were observed between knowledge and attitude (*r* = 0.412, *p* < 0.001), knowledge and practice (*r* = 0.273, *p* < 0.001), as well as attitude and practice (*r* = 0.403, *p* = 0.004) (Table 5).

**Table 5:** Pearson’s correlation analysis.

	Knowledge	Attitudes	Practices
Knowledge	1		
Attitudes	0.412 (*P* < 0.001)	1	
Practices	0.273 (*P* < 0.001)	0.403 (*P* < 0.001)	1

**Figure 1:**
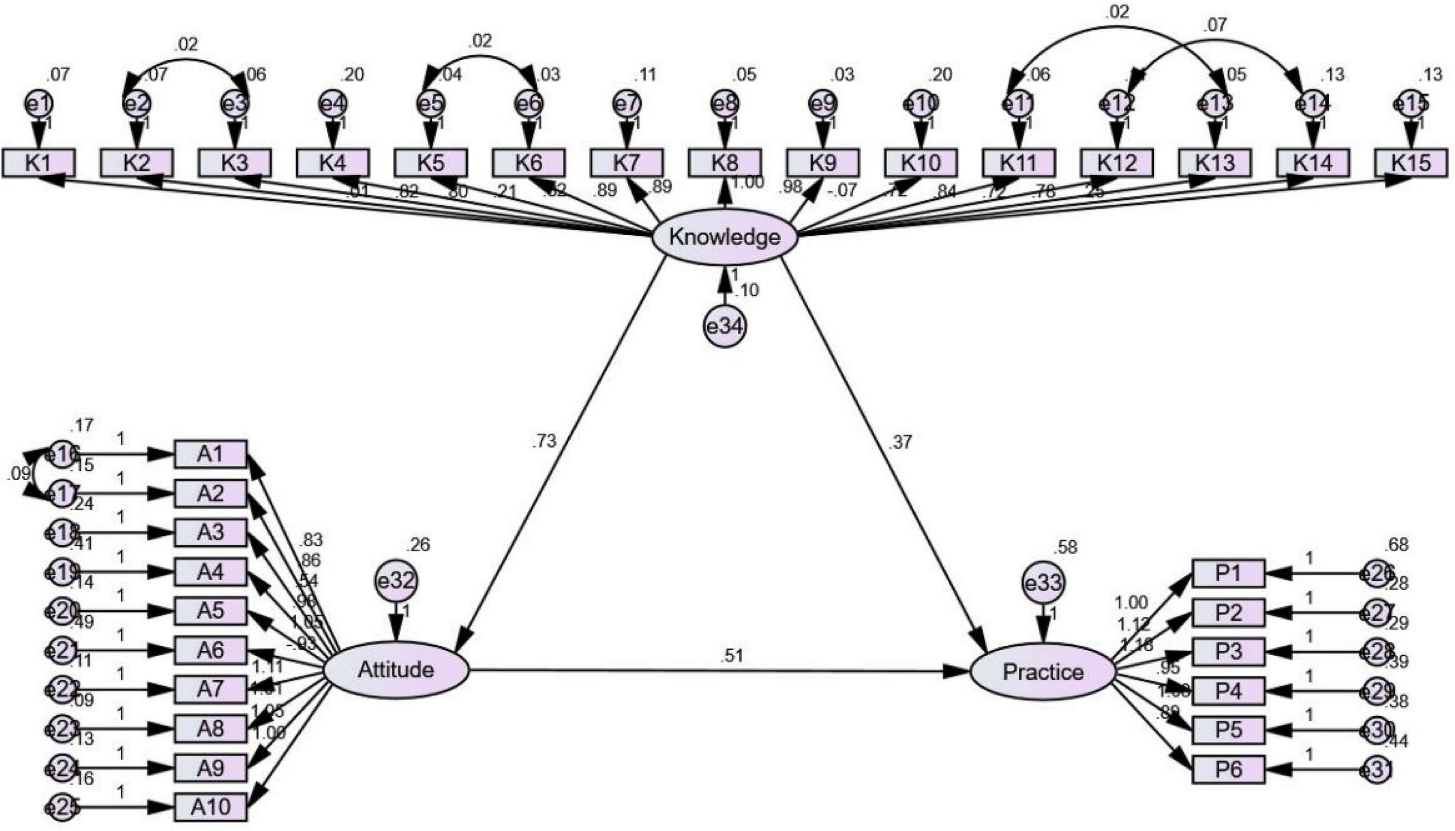
Structural equation modeling.

The SEM demonstrated that knowledge had direct effects on attitude and practice, as indicated by a path coefficient of 0.725 (*p* < 0.001) and 0.370 (*p* = 0.001), respectively. Furthermore, attitude had direct effects on practices, with a path coefficient of 0.509 (*p* < 0.001) (Table 6 and Figure 1). The fit indices of the model indicated a reasonably good fit to the data, implying an well fit for the proposed model (Table 7).

**Table 6:** Test results of the hypothesis.

Hypothesis			Estimate	*P*
Attitude	<−--	Knowledge	0.725	<0.001
Practice	<−--	Attitude	0.509	<0.001
Practice	<−--	Knowledge	0.370	0.001

**Table 7:** Model fitness indices for the KAP structural equation model.

Goodness-of-fit indices	Ideal standards	Measurement value
CMIN/DF	1–3 excellent, 3–5 good	3.750
RMSEA	<0.08 good	0.066
IFI	>0.8 good	0.910
TLI	>0.8 good	0.901
CFI	>0.8 good	0.910

CMIN/DF, Chi-square fit statistics/degree of freedom; RMSEA, root mean square error of approximation; IFI, incremental fix index; TLI, Tucker–Lewis index; CFI, comparative fix index.

## Discussion

The previous study revealed that medical students had sufficient knowledge, active attitude, and suboptimal practice toward management of depression. It is recommended to integrate practical application of depression-related knowledge into the curriculum, foster positive attitudes through empathy-building, and encourage peer learning and collaboration among medical students.

The study’s results indicate that various factors, including gender, academic grade, family medical background, place of residence, academic level, being the only child, and *per capita* income, had a significant impact on KAP scores. These findings are consistent with prior research and emphasize the need for tailored interventions in healthcare education and support (16, 17). The unexpected influence of academic grade on all three KAP domains suggests a need for further investigation to better understand its role and implications for healthcare behavior and education (18, 19). Misconceptions about depression among medical students can negatively impact patient care by leading to inadequate management and delayed interventions. These misunderstandings can result in insufficient patient education and engagement, ultimately hindering effective treatment and recovery. Correcting these misconceptions through targeted education is essential to improve patient outcomes.

This study assessed the knowledge of medical students regarding depression management, revealing a mix of correct and incorrect responses. Encouragingly, students recognized the importance of personalized medication approaches, comprehensive cognitive assessments, and the presence of comorbid anxiety disorders in depression patients. However, concerning misconceptions were found, such as the belief that cognitive symptoms in depression are limited to acute episodes, a preference for combination therapy, and underestimating the prevalence of major depressive disorder (20, 21). To enhance clinical practice, targeted educational interventions are needed to address these knowledge gaps, promote evidence-based practices, and ensure that medical students have accurate information for future patient care (22). Specific improvements could include integrating practical applications such as role-playing scenarios and case studies into the curriculum, fostering empathy through patient interaction and reflective exercises, and encouraging peer learning through group discussions and collaborative projects. Regarding attitude dimension, the results indicate that while medical students show a good grasp of depression’s insidious nature and potential severity, there’s room for improvement regarding their motivation to seek further information about depression and their perception of the practical value of related knowledge (3). To enhance clinical practice, it is essential to develop more targeted educational approaches that emphasize the clinical significance of depression-related knowledge and engage students more effectively in understanding its complexities (23). These measures will better equip future healthcare professionals to provide comprehensive care for individuals with depression, ultimately contributing to improved mental health outcomes.

The practice dimension indicated that while a relatively low percentage of medical students often engage in self-directed learning about depression, many demonstrate a proactive approach by participating in relevant courses and staying updated on assessment scales and guidelines (24). These practices suggest readiness to engage with the complexities of depression diagnosis and treatment, which is crucial for future clinical care. Furthermore, students generally endorse effective preventive measures against depression and express a willingness to help classmates suffering from depression, demonstrating a compassionate attitude that is vital for patient support (25). However, the presence of a small number of negative attitudes highlights the need for empathy-building programs within medical education to ensure that students are well-equipped for compassionate and effective patient care. Identified limitations in students’ practical skills further emphasize the necessity of integrating targeted interventions to bridge the gap between knowledge and practice. Specific improvements could include integrating practical applications such as role-playing scenarios and case studies into the curriculum, fostering empathy through patient interaction and reflective exercises, and encouraging peer learning through group discussions and collaborative projects. Future efforts should focus on fostering an environment of empathy, understanding, and open dialog, where individuals feel safe to seek help without fear of judgment. Collaboration among mental health professionals, policymakers, and communities is essential to eliminate the stigma associated with mental health, paving the way for improved mental health outcomes and a more compassionate society (26, 27).

The study’s correlation analysis and structural equation modeling reveal that knowledge, attitude, and practice regarding depression management among medical students are interconnected. Positive correlations between these factors indicate that as students acquire more knowledge about depression, they tend to develop more positive attitudes and engage in better practices. The structural equation modeling further highlights that knowledge has a direct effect on attitude and practice, emphasizing the central role of education in shaping attitudes and influencing clinical behavior. Additionally, a positive attitude directly influences practices, reinforcing the importance of cultivating a compassionate and empathetic approach within medical education (28). These findings underscore the need for a comprehensive and integrated approach to depression management education that addresses knowledge, attitudes, and practical skills to prepare future healthcare professionals for effective patient care.

This study had limitations. Firstly, this study’s reliance on a web-based survey may have introduced sampling bias, potentially limiting the representativeness of the participant sample. Additionally, self-reported data collection could have introduced recall and social desirability biases, potentially impacting the accuracy of responses. For future studies, it is recommended to explore the long-term effects of integrated depression management training on medical students’ clinical practice and patient outcomes. Additionally, examining the role of emerging digital tools and telemedicine in enhancing students’ KAP toward depression could provide valuable insights for modernizing healthcare education.

## Conclusion

The study suggests that while medical students possess a good level of knowledge and an active attitude toward depression management, their practical application of this knowledge remains suboptimal. Enhancing clinical practice among medical students should involve integrating practical application of depression-related knowledge, fostering empathy to promote positive attitudes, and promoting peer learning and collaboration.

## Data Availability

The original contributions presented in the study are included in the article/supplementary material, further inquiries can be directed to the corresponding author.

## Footnotes

^1^http://www.sojump.com
